# Synthesis, X-ray Structure, Hirshfeld, DFT and Biological Studies on a Quinazolinone-Nitrate Complex

**DOI:** 10.3390/molecules27031089

**Published:** 2022-02-06

**Authors:** Eman M. Fathalla, Mezna Saleh Altowyan, Jörg H. Albering, Assem Barakat, Morsy A. M. Abu-Youssef, Saied M. Soliman, Ahmed M. A. Badr

**Affiliations:** 1 Department of Chemistry, Faculty of Science, Alexandria University, P.O. Box 426, Ibrahimia, Alexandria 21321, Egypt; Eman.nomeir@alexu.edu.eg (E.M.F.); ahmed_badr@alexu.edu.eg (A.M.A.B.); 2 Department of Chemistry, College of Science, Princess Nourah bint Abdulrahman University, P.O. Box 84428, Riyadh 11671, Saudi Arabia; msaltowyan@pnu.edu.sa; 3 Graz University of Technology, Mandellstr. 11/III, A-8010 Graz, Austria; joerg.albering@tugraz.at; 4 Department of Chemistry, College of Science, King Saud University, P.O. Box 2455, Riyadh 11451, Saudi Arabia; ambarakat@ksu.edu.sa

**Keywords:** quinazolinone-nitrate, isomers’ stability, Hirshfeld, DFT, antioxidant, anticancer, antimicrobial

## Abstract

The reaction of 4-hydroxyquinazoline (**4HQZ**) with aqueous solution of nitric acid afforded the corresponding quinazolinone-nitrate (**4HQZN**) complex in very good yield. The crystal structure of **4HQZN** was determined and its structural and supramolecular structural aspects were analyzed. **4HQZN** crystallized in the space group P2_1_/c and monoclinic crystal system with one [**4HQZ**-H]^+^[NO_3_]^−^ formula and Z = 4. Its supramolecular structure could be described as a 2D infinite layers in which the **4HQZN** molecules are connected via N-H…O and C-H…O hydrogen bridges. Using DFT calculations, the relative stability of five suggested isomers of **4HQZN** were predicted. It was found that the medium effects have strong impact not only on the isomers’ stability but also on the structure of the **4HQZN**. It was found that the structure of **4HQZN** in DMSO and methanol matched well with the reported X-ray structure which shed the light on the importance of the intermolecular interactions on the isomers’ stability. The structure of **4HQZN** could be described as a proton transfer complex in which the nitrate anion acting as an e-donor whiles the protonated **4HQZ** is an e-acceptor. In contrast, the structure of the isolated **4HQZN** in gas phase and in cyclohexane could be described as a **4HQZ…HNO_3_** hydrogen bonded complex. Biological screening of the antioxidant, anticancer and antimicrobial activities of **4HQZ** and **4HQZN** was presented and compared. It was found that, **4HQZN** has higher antioxidant activity (IC_50_ = 36.59 ± 1.23 µg/mL) than **4HQZ**. Both of **4HQZ** and **4HQZN** showed cell growth inhibition against breast (MCF-7) and lung (A-549) carcinoma cell lines with different extents. The **4HQZ** has better activity with IC_50_ of 178.08 ± 6.24 µg/mL and 119.84 ± 4.98 µg/mL, respectively. The corresponding values for **4HQZN** are 249.87 ± 9.71 µg/mL and 237.02 ± 8.64 µg/mL, respectively. Also, the antibacterial and antifungal activities of **4HQZN** are higher than **4HQZ** against all studied microbes. The most promising result is for **4HQZN** against *A. fumigatus* (MIC = 312.5 μg/mL).

## 1. Introduction

**Figure 1:**
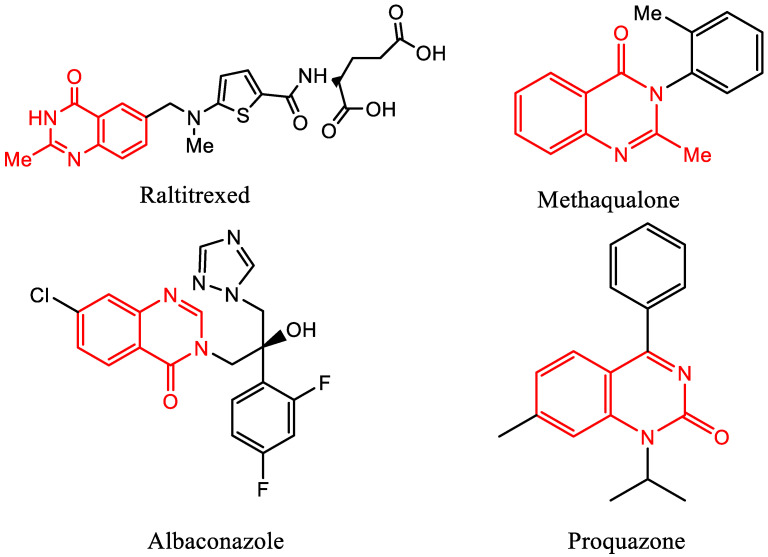
Structure of some quinazoline/quinazolidinone-based medicines.

In organic chemistry, 5- or 6-membered rings containing oxygen, nitrogen, or sulphur atom are the most abundant natural heterocycles [1,2]. Quinazoline (1,3-diazanaphthalene) is a nitrogen-containing fused heterocycle [3,4] consisting of two fused 6-membered simple aromatic rings: a benzene and a pyrimidine [5,6]. Quinazolines have a broad range of pharmacological applications, in addition to their low toxicity profiles [3]. Quinazoline frameworks have great pharmacological activities as antioxidant [7,8,9], antidiabetic [10,11,12], antibacterial [13,14,15], antiviral [16,17,18], antimicrobial [19,20,21], anticancer [22,23], antihistaminic [24,25,26], antidiuretic [27,28], antitubercular [29,30,31], and anti-inflammatory [32,33,34] agents. Quinazolinones are a type of quinazoline derivative that are biologically active in the same way as quinazoline [35]. There are various medications that include quinazoline and quinazolinone rings, such as raltitrexed, a quinazolinone-based medicine, which is used to treat large intestine cancer, whereas methaqualone is a sedative drug, albaconazole is an antifungal drug and proquazone is a non-steroidal anti-inflammatory drug (Figure 1) [36,37,38,39].

**Figure 2:**
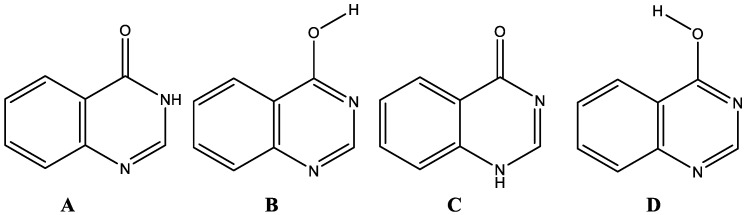
Isomeric forms of 4-hydroxyquinazoline (**4-HQZ**).

There has been a lot of effort in investigating the tautomerism of heterocyclic systems during the past two decades in order to figure out how tautomerism affects molecules chemically and biologically. Understanding the varied features of tautomers would require a careful investigation of the structure and changes in both geometrical and energetic variables produced via the transfer of hydrogen atoms. From the standpoint of structural chemistry, understanding the comparative stabilities of the tautomers or isomers of heterocycles, and even the transformation between different isomeric forms, is a critical [40]. Environmental factors such as solvent polarity are extremely important in heterocyclic tautomeric equilibria [41,42,43,44,45,46,47,48]. In this regard, 4-hydroxyquinazoline (**4-HQZ**) could exist as the four possible isomers as shown in Figure 2. In 2011 Polat et al. [49], reported the properties of structure and energy of the different isomers of **4-HQZ**. They reported that the most stable form is tautomer **A** in both the gaseous and aqueous phases based on DFT calculations, and the sequence of tautomer stability in the gas phase is **A > B > C > D**.

**Figure 3:**
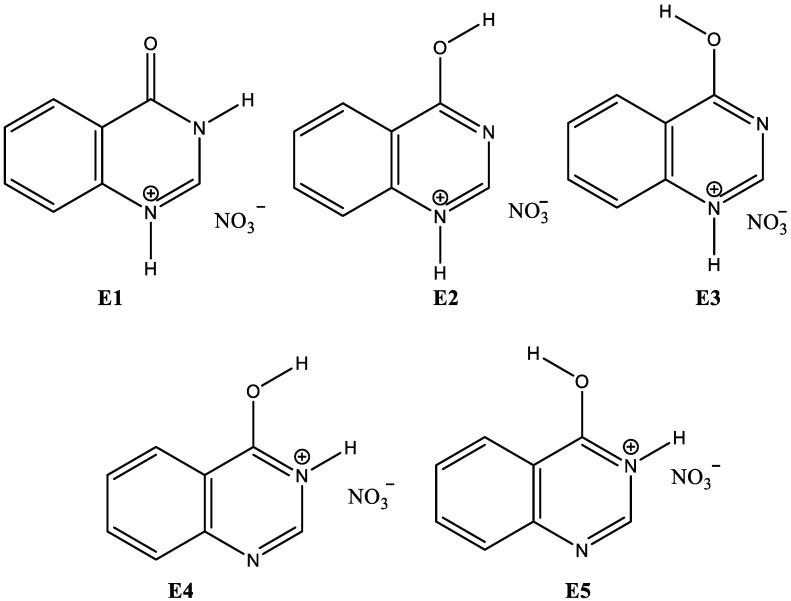
Isomeric forms of **4HQZN**.

Given the relevance of the quinazoline derivatives, we report here the synthesis of the nitrate salt of **4HQZ** (**4HQZN**). The structure of the **4HQZN** was characterized using elemental analysis and FTIR spectroscopy as well as single crystal X-ray diffraction. Its supramolecular structure aspects were analyzed using Hirshfeld calculations. Also, a detailed examination for the stability of all potential isomers of **4HQZN** (Figure 3) in various media such as methanol, DMSO and cyclohexane was performed using DFT quantum chemical simulations. Finally, we examined the antioxidant, anticancer and antimicrobial activities of **4HQZN** compared to the free **4HQZ**.

## 2. Results and Discussion

### 2.1. Synthesis and Chracterizations

The compound **4HQZN** was synthesized using the direct reaction between **4HQZ** and nitric acid. The structure of **4HQZN** was identified using elemental analysis, FTIR and single crystal X-ray diffraction. Similar to the free **4HQZ**, the corresponding salt **4HQZN** could exist in the five isomeric forms shown in Figure 3. X-ray structure analysis revealed with no doubt the presence of the **4HQZN** in the form **E1** in the solid crystalline form. In this regard, comparative studies for the stability of the different isomeric forms of **4HQZN** in gas phase and in solution were elucidated using DFT calculations. In addition, the supramolecular structural details of **4HQZN** were also investigated based on Hirshfeld calculations. Its bioactivity as antioxidant, anticancer and antimicrobial agent were determined and compared with the free **4HQZ**.

### 2.2. Structure Description

**Figure 4:**
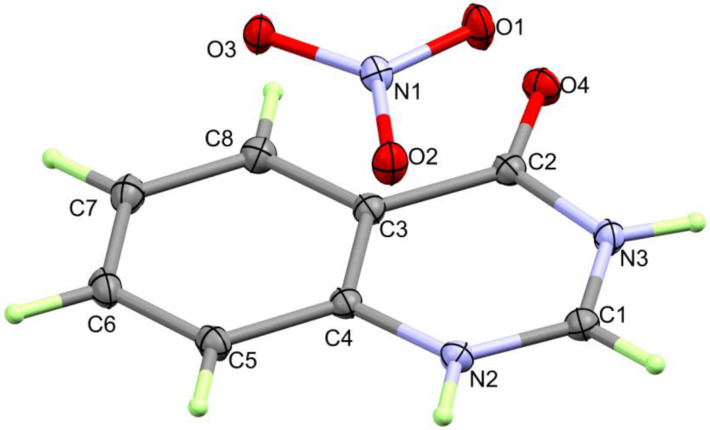
Structure of **4HQZN**.

The compound **4HQZN** crystallizes in the space group *P2_1_/c* with one formula unit of the salt [**4HQZ**-H]^+^[NO_3_]^−^ per asymmetric unit. The elementary cell contains a total of *Z* = 4 formula units (Table S1; Supplementary Materials). The interatomic distances and bond angles—except those involving hydrogen atoms can be taken from Table 1. Figure 4 shows one cation and one anion of the structure of **4HQZN**.

The carbon atoms of the benzene ring (C3C4C5C6C7C8) show the interatomic distances expected for a delocalized π-electron system in the range from 1.3838(14) Å (C7-C8) to 1.4004(13) Å (C3-C8). The bond angles in this ring system also deviate only slightly from the ideal value of 120° (C6-C5-C4: 118.65(9)° and C5-C4-C3: 121.30(9)°). The bond distance between C2 and C3 of 1.4652(13) Å shows by its value between those for a typical single and a double bond that the carbonyl group is also involved in the π-electron system of the benzene ring. The bond length of C2-O4: 1.2141(12) Å found for the carbonyl group is within the normal range. All 11 atoms of the two ring systems of the cation deviate very little from planarity. The largest deviation occurs with the atom N2, which lies 0.039 Å below the averaged ring plane. The carbonyl oxygen, on the other hand, shows a deviation of 0.032 Å in the opposite direction. The plane of the amide group thus deviates by about 1.9° from the averaged ring plane of the entire cation.

The electron density map of the structure clearly shows that both nitrogen atoms are protonated. For the structure refinement, their B parameters were fixed to 1.5 times the values for respective nitrogen atoms, and the position was refined using the DFIX command in SHELX. Both N-H bond lengths are in the expected range: N2-H2 0.914(14) Å and N3-H3 0.875(15) Å. What has already been said about the organic cation in general also applies to the nitrate anion: no significant deviations occur with respect to the bond lengths and bond angles. All values can be taken from Table 1 and are within the normal range. Furthermore, there are no unusual deviations of the vibrational ellipsoids from the ideal spherical shape, neither for the organic cation nor for the nitrate anion.

**Figure 5:**
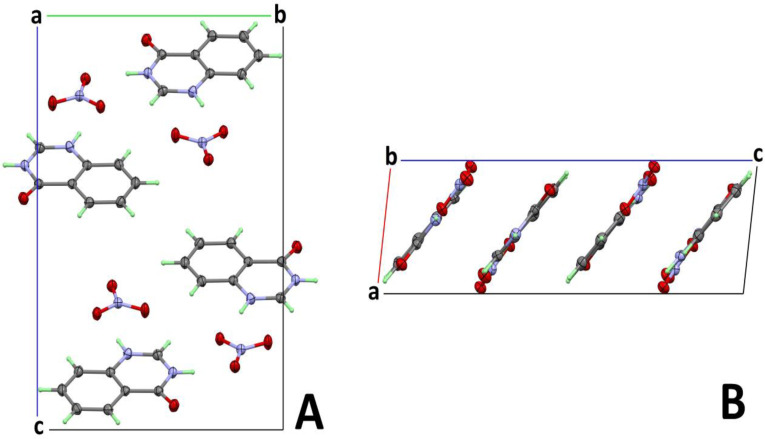
Packing scheme of **4HQZN**. Part (**A**) shows a projection along 100, part (**B**) along the monoclinic *b*-axis 010.

**Figure 6:**
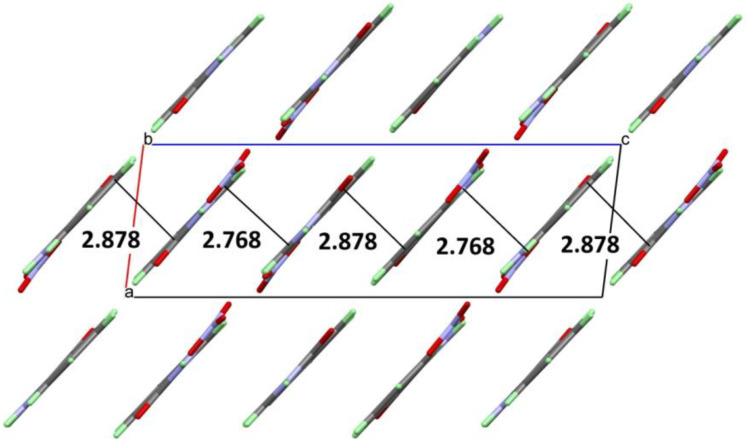
View of layered packing along *b*-axis. The number indicated the alternating layer distances in (Å).

The packing scheme of **4HQZN** has a distinct layered structure. This can be seen particularly well in Figure 5B and Figure 6. Four layers of almost co-planar organic cations and nitrate ions cross the elementary cell parallel to the crystallographic *b*-axis. A closer look reveals that the molecular planes of anions and cations are not completely co-planar, but adopt an angle of 6.2° with respect to each other. This leads to a very weak corrugation of the layers.

**Figure 7:**
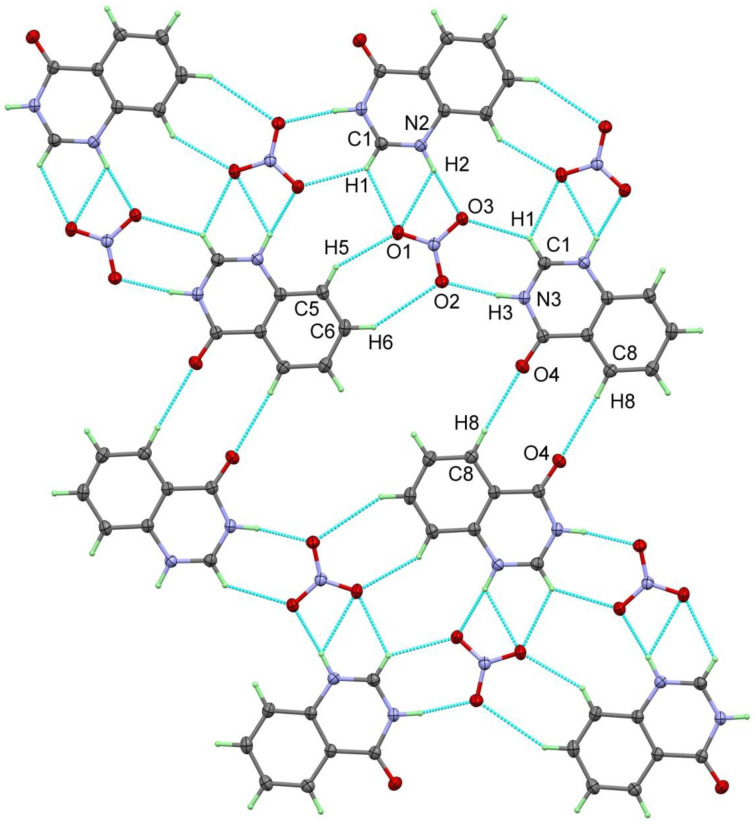
Hydrogen bridges in the 2-dimensional infinite layers occurring in the crystal structure of **4HQZN**. All hydrogen bridges are presented as light blue, broken lines. Hydrogen bond parameters are listed in Table 2.

Figure 7 shows the 2-dimensional infinite layers of alternately arranged anions and cations, which are linked by numerous hydrogen bonds. The layers defined by the approximately co-planar organic cations and the nitrate anions have two slightly different mean distances to each other of 2.768 Å and 2.878 Å, as shown in Figure 6. This is probably due to the fact that the ionic interactions between the layers are slightly different. These interactions are much stronger than the few π-π contacts of the organic molecules, and thus the ionic interactions dominate the formation of the comparatively short layer distances (for pure π-π stacking, distances in the range of about 3.2–3.4 Å would be common).

The 2-dimensional infinite layers in the structure of quinazolinone nitrate are composed of approximately co-planar aligned organic cations and nitrate anions in 1-dimensional infinite “bands”, approximately 11 Å wide, which are themselves infinitely linked in the second dimension via further weak hydrogen bonds through the carbonyl oxygen atoms O4 and the hydrogen H8 bound to C8 in the C6 ring system. Within the broad “bands”, nitrate ions arranged in a zig-zag pattern form the inner part, i.e., to a certain extent the analog of a backbone of a coordination polymer. Each nitrate ion is linked via its oxygen atoms twice (O2 and O3) or three times (O1) to protons of three neighboring organic cations via hydrogen bonds. The strongest interactions are N2-H2…O3 and N3-H3…O2 with donor-acceptor distances of 2.7943(11) Å and 2.7790(11) Å, respectively. Other hydrogen bonds with significantly longer contact distances are found for C1-H1…O3 (3.0961(12) Å), C1-H1…O1 (2.9920(13) Å), C5-H5…O1 (3.1953(13) Å), and for N2-H2…O1 (2.9950(12) Å). The linkage of the “bands” occurs through C8-H8…O4 interactions and relatively weak O…H contacts (2.50 Å). In spite of these subtle distinctions, one can rightly consider the layers as two-dimensional infinite.

### 2.3. Hirshfeld Analysis

**Figure 8:**
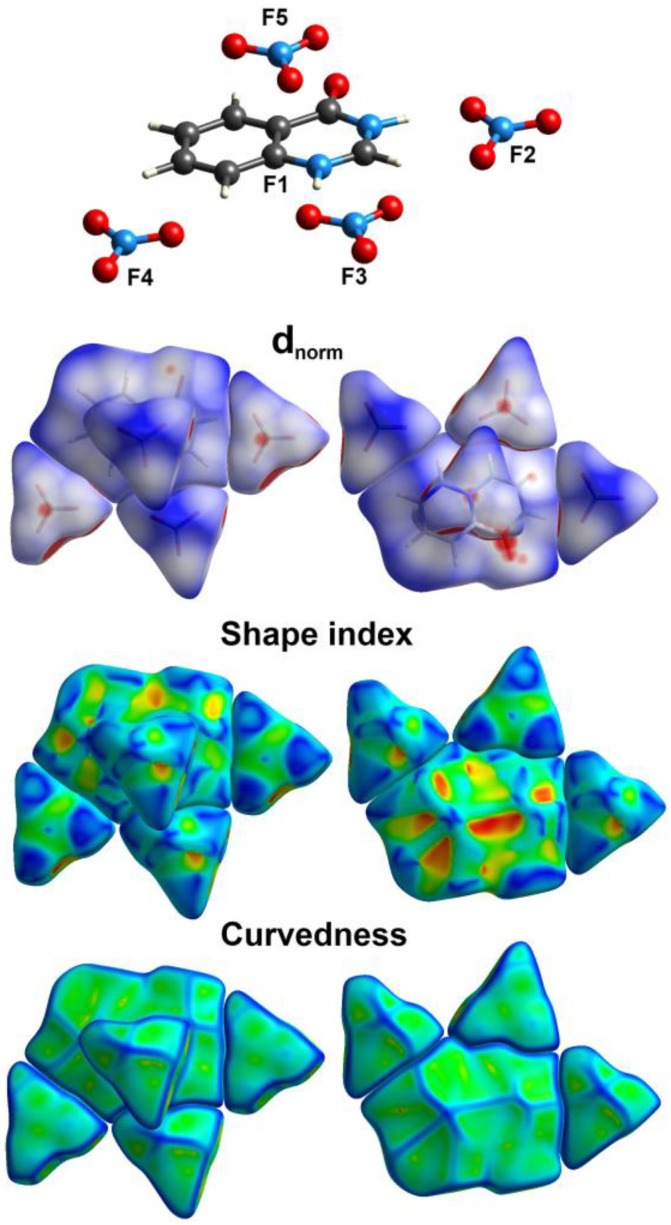
Hirshfeld surfaces mapped over shape index (SI), curvedness as well as d_norm_ for the studied five fragments (**F1** to **F5**) of **4HQZN**.

The stability of the studied crystal is greatly determined by the intermolecular interactions occurring in it, most of these interactions could be quantitatively analyzed using Hirshfeld calculations. In the crystal structure of **4HQZN**, there are four nitrate anions surrounding the protonated **4HQZ**. To shed more insights on the intermolecular interactions in the crystal structure of **4HQZN**, we performed the Hirshfeld analysis for the five fragments. The resulting Hirshfeld surfaces are presented in Figure 8.

**Figure 9:**
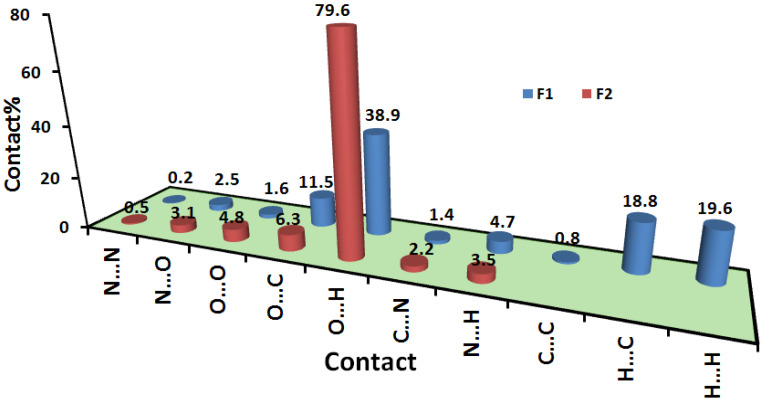
Intermolecular interactions in the protonated **4HQZ** (**F1**) and nitrate (**F2**) fragments. Hirshfeld analysis of the four nitrate fragments gave the same results, hence only one is presented.

**Figure 10:**
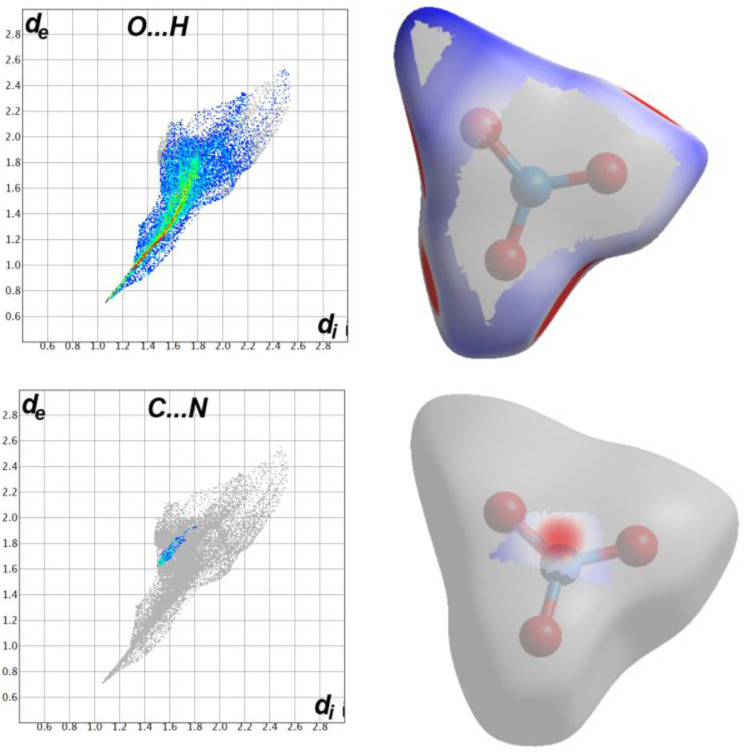
Hirshfeld analysis of the nitrate fragment (**F2**).

Analysis of the fingerprint plots of these fragments indicated with no doubt that the four nitrate anion fragments have exactly the same pattern of intermolecular contacts as well as the same contributions for each contact. Hence, the contribution of these intermolecular contacts in only one fragment is presented in Figure 9. The most dominant contacts are the O…H interactions which contributed by 79.6% from the whole area of the fingerprint of the nitrate anion fragment (**F2**). In the d_norm_ map of the nitrate anion, there are a number of red spots corresponding to the O…H hydrogen bonds with the protonated **4HQZ** cationic part (**F1**) where the O3…H2 (1.787 Å) is the shortest. In addition, there is another red spot related to the short C4…N1 (3.134 Å) contact (Figure 10). The latter contributed by only 2.1% from the whole fingerprint area.

**Figure 11:**
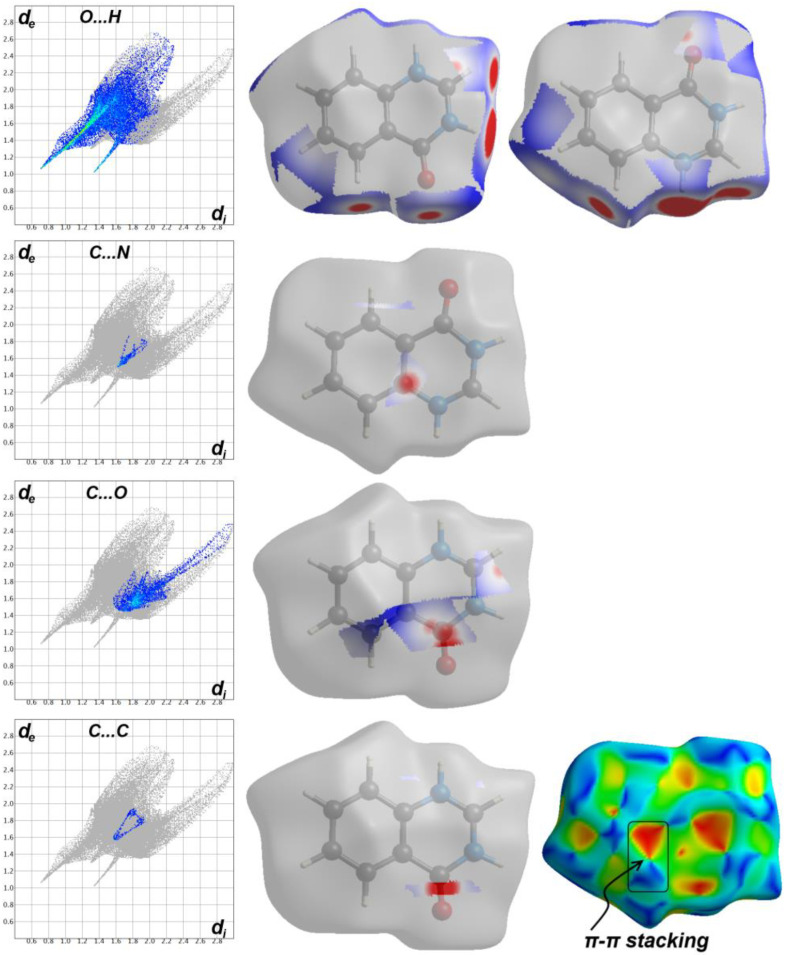
Hirshfeld analysis of the protonated **4HQZ** fragment (**F1**).

The d_norm_ and fingerprint of the most important intermolecular contacts in the protonated **4HQZ** fragment (**F1**) are summarized in Figure 11. It is clear that the O…H (38.9%), H…H (19.6%), C…H (18.8%) and C…O (11.5%) contacts represent the greatest contribution to the overall molecular packing interactions especially O…H hydrogen bonds. In addition to the short O3…H2 and C4…N1 interactions, the C…C (0.6%) and C…O contacts contribute significantly to the stability of the crystalline structure and appear in the d_norm_ map as red spots which indicate intermolecular interactions between atoms have shorter distances than sums of their vdW radii, while the white or blue areas refer to the less important interactions.

The significantly short O…H, C…O, C…N and C…C distances are collected in Table 3. In addition, the presence of short C2…C2 interaction between two staked aromatic ring system is indicative for little π-π stacking interactions due to the misfit of the two stacked ring from one another. Hence, the shape index map showed the characteristic red/blue triangles for π-π stacking interactions (Figure 11).

### 2.4. DFT Studies

#### 2.4.1. Energetics and Stability

**Figure 12:**
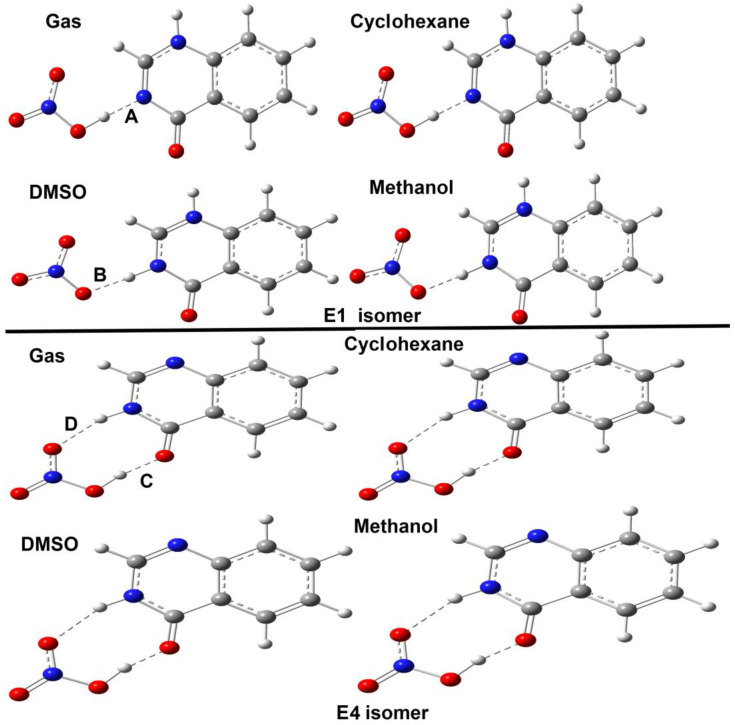
Optimized structures of the most stable isomers of **4HQZN** in different solvents. The intramolecular hydrogen bond contact distances are depicted in Table 5.

The studied compound **4HQZN** could exist in the five possible isomers shown in Figure 3. Based on the energies and thermodynamic parameters presented in Table 4, their relative stability was predicted (Table 4). It was found that the isomer **E4** has the lowest energy compared to the other isomers in the gas phase. Hence, **E4** is the most stable one in that phase. The isomer **E1** has higher energy than **E4** by 9.607 kcal·mol^−1^ and is considered the second most stable form. Also, **E4** is more stable than **E1** by 6.534 kcal·mol^−1^ in a nonpolar solvent (cyclohexane). Hence, the most stable isomer in these cases is not the same as the one observed i X-ray single crystal structure. The higher stability of **E4** compared to **E1** could be attributed to the presence of two intramolecular hydrogen bonding interactions in the former while the latter is stabilized by only one intramolecular hydrogen bond (Figure 12). In accord with these results, the thermodynamic parameters indicated that isomer **E4** is thermodynamically the most stable one in gas phase and in the nonpolar solvent cyclohexane (Table 4). In these medium, the order of the isomers’ stability is **E4** ˃ **E1** ˃ **E2** ˃ **E3** ˃ **E5**.

In contrast, the most stable isomer in methanol and DMSO as examples for polar protic and aprotic solvents, respectively, is **E1** while the second most stable isomer is **E4**. The latter is energetically higher by 2.748 and 3.034 kcal·mol^−1^ than the former in methanol and DMSO, respectively. Interestingly, this observation agrees very well with the X-ray structure of **4HQZN**. The question is why the situation is reversed in polar solvents? It is clear that the presence of intermolecular interactions with polar solvent molecules such as DMSO or methanol stabilized the **E1** isomer in solution. Similarly, the intermolecular interactions occurred among the molecular units of **4HQZN** molecules in the crystalline solid state structure have the upper hand on the existence of the **4HQZN** in the form **E1**. As a result, the most stable isomer in polar solvents was found to be **E1** which has similar situation to that in the solid state. In contrast, the intermolecular interactions in gas phase or in nonpolar solvent (cyclohexane) are neglected or almost weak, respectively. Hence, the intramolecular interactions are the dominant. As a result, the most stable isomer in these cases is **E4** due to the presence of two intramolecular hydrogen bonding interactions instead of one in **E1** (Table 5). These results shed the light on the importance of intra and inter-molecular interactions on the isomer’ stability.

It is obvious that the structure of **4HQZN** in gas phase and cyclohexane comprised of a **4HQZ** molecule hydrogen bonded with the HNO_3_ without a significant proton transfer where the O-H…N hydrogen bond distances in these cases are 1.640 and 1.563 Å, respectively. Hence, the structure of **4HQZN** could be simply described as **4HQZ…HNO_3_** hydrogen bonded complex. In contrast, there is a significant proton transfer in the case of polar solvents such as DMSO and methanol where the **4HQZ** seems to be protonated and the N-H…O hydrogen bond distances are 1.664 and 1.661 Å, respectively. In these cases, the structure is better to be described as a proton transfer complex of [**4HQZ**-H]^+^[NO_3_]^−^.

#### 2.4.2. Optimized Geometry

The optimized bond distances and angles of the **4HQZN** (isomer **E1**) in different solvents compared to the experimental X-ray structure results are listed in Table S2 (Supplementary Materials). There is no doubt about the better agreement between the calculated geometric parameters in DMSO and methanol with the X-ray crystal structure than those obtained from the optimized geometry in gas phase or in cyclohexane. It is clear that, the labile proton H3 is bonded with the more electronegative oxygen atom O2 of nitrate ion in both gas phase and in cyclohexane where the electrostatic interactions are predominant in these cases (Figure 12). The O2-H3 bond distances are predicted to be 1.033 and 1.055 Å in gas phase and cyclohexane, respectively. In contrast, in case of solutions of polar solvents like methanol and DMSO this proton (H3) appears to behave differently where these solvents effectively solvate the O-atoms of the NO_3_^−^ ion leading to a proton transfer to the nitrogen atom of the **4HQZ** moiety as indicated from the short N3-H3 bond which agreed with the X-ray structure. The calculated N-H distances are 1.059 and 1.058 Å in methanol and DMSO, respectively.

#### 2.4.3. Frontier Molecular Orbitals (FMOs) and Reactivity Descriptors

The HOMO and LUMO levels are important for understanding the intramolecular charge transfer [50,51]. Their energies are given in Table 6. It is clear that, the HOMO level is destabilized while LUMO level is stabilized in presence of polar solvents compared to that in the gas phase or in cyclohexane. As a consequence of the smaller decrease in the E_LUMO_ compared to the increase in the E_HOMO_, the values of the HOMO-LUMO energy gap are less in polar solvents compared to those in the gas phase or in cyclohexane.

**Figure 13:**
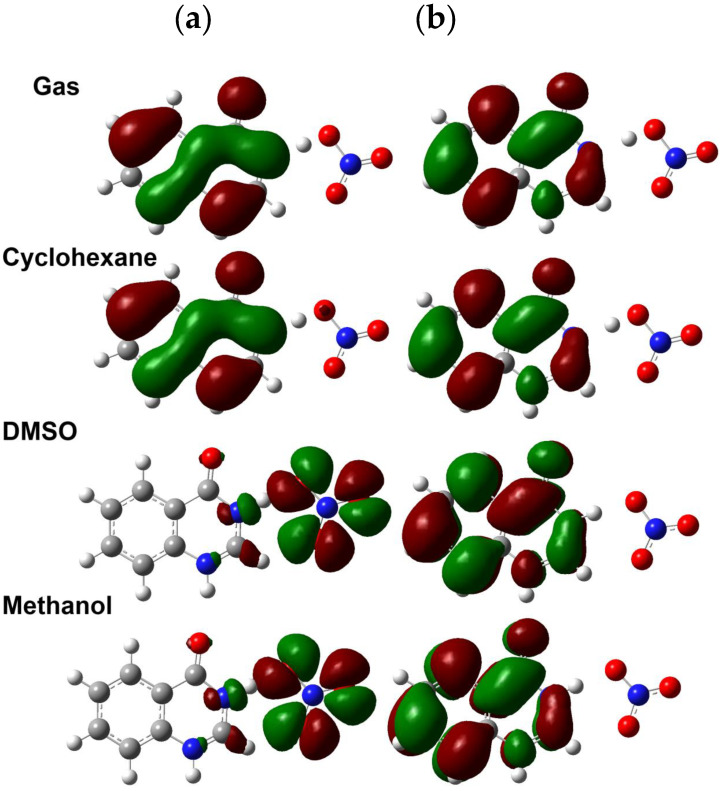
FMOs (HOMO; (**a**) and LUMO; (**b**)) in gas phase, cyclohexane, DMSO and methanol, from up to down, respectively.

Presentation of the HOMO and LUMO levels of **4HQZN** in different solvents is shown in Figure 13. In case of the hydrogen bonded system **HNO_3_…4HQZ**, the optimized structure of **4HQZN** in gas phase and in cyclohexane, both HOMO and LUMO levels are delocalized over the **4HQZ** π-system. Hence, the HOMO → LUMO excitation could be considered as intramolecular excitation within the **4HQZ** moiety. In contrast, the HOMO level of the proton transfer complex **4HQZN** at the optimized structure in DMSO and methanol is found located over the NO_3_^−^ moiety while the LUMO level is delocalized over the protonated quinazoline ring π-system indicating an intermolecular HOMO → LUMO excitation. These differences could be main reasons for the variations in the HOMO-LUMO energy gap with the nature of solvent.

#### 2.4.4. Natural Charge Population

Natural atomic charges were calculated in order to predict the extent of the charge transfer within the different structures of the isomer **E1** depending on the nature of solvent. The results of the natural charges are listed in Table S3 (Supplementary Materials). In case of the **4HQZ…HNO_3_** hydrogen bonded system, the net charges at the **4HQZ** moiety are predicted to be 0.093 e and 0.121 e in gas phase and cyclohexane, respectively. In contrast, the net charges at the HNO_3_ fragment are −0.093 e and −0.121 e, respectively. The corresponding value in the isolated fragments is zero. Hence, the **4HQZ** acting as an electron donor whiles the HNO_3_ fragment is an electron acceptor. For the proton transfer complex **4HQZN**, the net charges at the protonated **4HQZ** are 0.898 and 0.897 e, in DMSO and methanol, respectively instead of +1 e. On the other hand, the net charges at the nitrate ion are −0.898 and −0.897 e, respectively, instead of −1 e. Hence, the latter acting as an electron donor whiles the former is the electron acceptor. The amounts of electrons transferred in these cases are 0.101 and 0.103 e, respectively. At the X-ray geometry, the corresponding value is 0.063 e.

### 2.5. Biological Studies

#### 2.5.1. Antioxidant Activity

**Figure 14:**
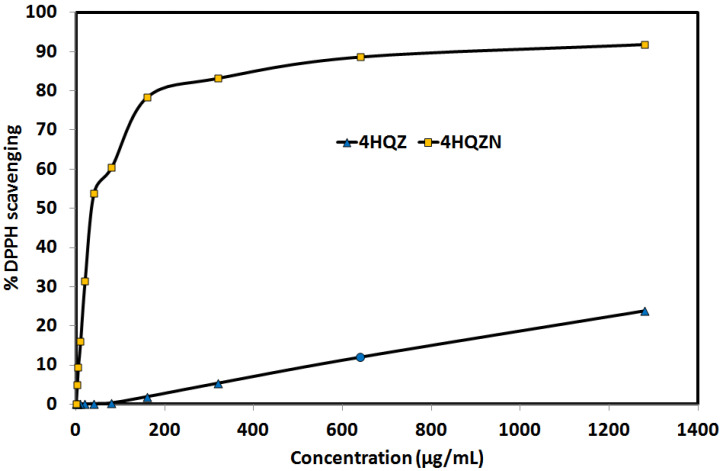
The antioxidant activity of **4HQZ** and **4HQZN**.

The DPPH [52,53] free radical scavenging assay enabled us to determine the antioxidant activity of **4HQZ** and its nitrate salt **4HQZN** (Tables S4 and S5; Supplementary Materials). For better clarity, the results are summarized graphically in Figure 14. It is clear that the antioxidant activity of **4HQZ** is weak. The DPPH scavenging is only 23.76% at 1280 µg/mL compared to 91.89% for **4HQZN** at the same concentration. Hence, the **4HQZN** has higher antioxidant activity than **4HQZ** and the former has IC_50_ value of 36.59 ± 1.23 µg/mL. In this regard, the **4HQZ** has moderate antioxidant activity in comparison with ascorbic acid as reference standard (IC_50_ = 10.62 ± 0.78 µg/mL). It is clear that the presence of the nitrate ion could have a direct impact on the enhancement of the antioxidant activity of **4HQZN**. In agreement with literature, the nitrate supplementation has the ability to operate as an antioxidant, inhibiting the generation of free radicals [54] which is found consistent with the higher antioxidant activity of **4HQZN** than **4HQZ**.

#### 2.5.2. Cytotoxicity against Breast and Lung Carcinoma

**Figure 15:**
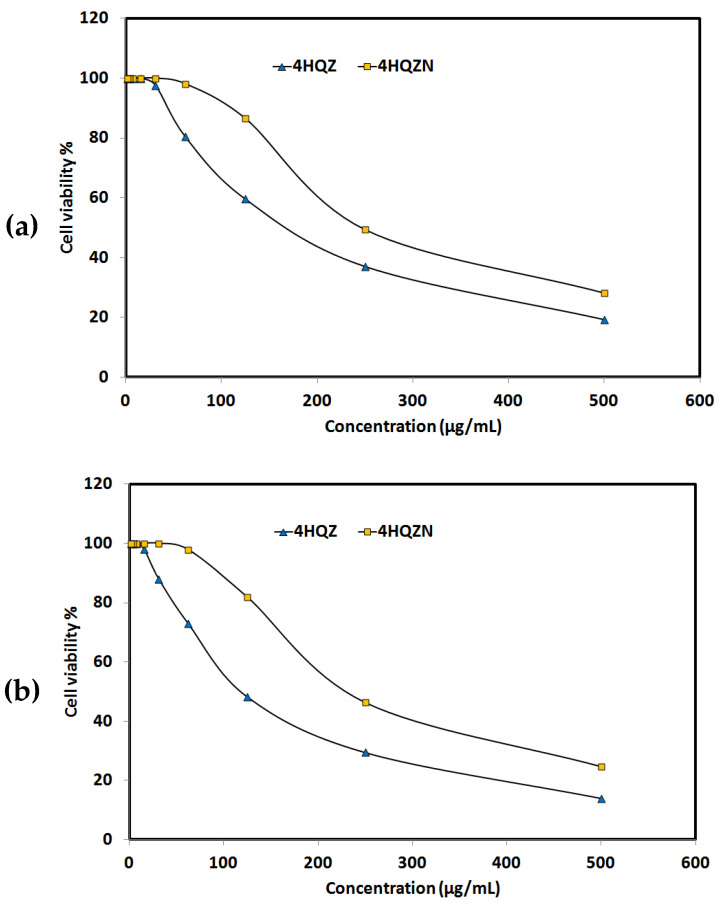
The cytotoxic activity of **4HQZ** and **4HQZN** against breast (**a**) and lung carcinoma (**b**).

The anticancer activities of the **4HQZ** and **4HQZN** against breast (MCF-7) and lung (A-549) carcinoma cell lines were examined. The cell viability values are listed in Tables S6–S9 (Supplementary Materials), respectively, and presented graphically in Figure 15 (upper part). Both of **4HQZ** and **4HQZN** showed cell growth inhibition against breast carcinoma MCF-7 cell line to different extents. Generally the results indicated that **4HQZ** has better activity against breast carcinoma MCF-7 cell line with IC_50_ of 178.08 ± 6.24 µg/mL than **4HQZN**. The IC_50_ of the latter is higher (249.87 ± 9.71 µg/mL) indicating lower activity against breast carcinoma. In addition, the anticancer activity of the **4HQZ** and **4HQZN** against lung carcinoma A-549 cell line were also evaluated (Figure 15, lower part). Also, both compounds showed weak to moderate anticancer activity with IC_50_ values of 119.84 ± 4.98 and 237.02 ± 8.64 µg/mL. The lower IC_50_ values of the former compared to the latter indicated the better anticancer activity of **4HQZ** than **4HQZN** against this cell line.

#### 2.5.3. Antimicrobial Activity

Different categories of microbes were used to examine the antimicrobial activity of **4HQZ** and **4HQZN** by determining the minimum inhibition zone diameters against these microbes at 10 mg/mL (Table 7).

The results shown in Table 7 leave no doubt about the antibacterial and antifungal activities of both compounds against the studied microbes. The only exception is **4HQZ** which is found inactive against *A. fumigatus* at the applicable concentration while the inhibition zone diameter for **4HQZN** is determined to be 18 mm. Generally, the inhibition zone diameters of the nitrate complex **4HQZN** are larger for all microbes compared to the **4HQZ**. Hence, one could conclude the higher antibacterial and antifungal activities of **4HQZN** than **4HQZ** against all the investigated microbes. The inhibition zone diameters range from 9 mm (*C. albicans* and *B. subtilis*) to 11 mm (*E. coli* and *P. vulgaris*) for **4HQZ**. The corresponding values for **4HQZN** are 10 mm (*C. albicans*) and 18 mm (*A. fumigatus*). Generally, both compounds could be considered as a broad-spectrum antimicrobial agent against the selected Gram positive and Gram negative bacterial strains and the fungus *C. albicans* as well.

The Minimum Inhibitory Concentrations (MIC, in μg/mL) presented in Table 8 indicated higher potency of **4HQZN** than **4HQZ** against all microbes. For all microbes (except *B. subtilis*), the **4HQZN** has lower MIC values than **4HQZ**. The former has higher potency against the fungus *A. fumigatus* and *C. albicans* as well as the bacteria *S. aureus*, *E. coli* and *P. vulgaris* than the latter. The most promising result is for **4HQZN** against *A. fumigatus* with MIC value of 312.5 μg/mL compared to 156.25 μg/mL for Ketoconazole. The rest of MIC values are significantly higher than those for the standard drugs ketoconazole and gentamycin as antifungal and antibacterial drugs, respectively.

## 3. Experimental

### 3.1. Materials and Physical Measurements

All chemicals including 4-hydroxyquinozaline (**4HQZ**) were purchased from Sigma-Aldrich Chemical Company Inc. (St. Louis, MO, USA). Elemental analyses (CHN) were measured using a model 2400 instrument (Perkin-Elmer, city, state abbreviation if USA, country). The FTIR spectra were measured at 4000–400 cm^−1^ using a Tensor 37 FTIR instrument (Bruker, city, state abbreviation if USA, country) in KBr pellets.

### 3.2. Synthesis of **4HQZN**

**4HQZN** was obtained by mixing 0.292 g (2 mmol) of **4HQZ** in ethanol with 1 mL of 0.2 mol/L nitric acid (2 mmol). A white precipitate was immediately obtained which was then dissolved in acetonitrile. After five days, pure colorless needle crystals of the target compound **4HQZN** were obtained. Yield; C_8_H_7_N_3_O_4_ 87%; m.p. = 194–196 °C. Anal. Calc. C, 45.94; H, 3.37; N, 20.09%. Found: C, 45.81; H, 3.30; N, 20.17%. IR (KBr, cm^−1^): 3417, 3099, 3067, 1659, 1609, 1718, 1385 (Figure S1, Supplementary Materials).

### 3.3. Crystal Structure Determination

The determination of the crystal structure of **4HQZN** was performed using the procedures described in the Supplementary Materials [55,56].

### 3.4. Biological Studies

The antimicrobial activity of **4HQZ** and **4HQZN** against different microbes and their antioxidant activity were performed according the procedures mentioned in Methods S1 and S2 (Supplementary Materials). Also, evaluation of cytotoxic activities against breast MCF-7 and lung A-549 carcinoma cell lines were performed following the procedure mentioned in Method S3.

### 3.5. Computational Details

Crystal Explorer 17.5 program [57] was used for Hirshfeld analysis. The structures of the studied isomers shown in Figure 3 were calculated using Gaussian 09 program [58,59,60,61,62]. Further details are found in Supplementary Materials.

## 4. Conclusions

The molecular and supramolecular structure aspects of **4HQZN** were investigated using different experimental and theoretical procedures. The crystal structure of **4HQZN** was analyzed using topology analysis with the aid of Hirshfeld calculations. Its supramolecular structure could be described as a 2D infinite layers of hydrogen-bonded **4HQZN** molecules. Five possible isomers of **4HQZN** were calculated using DFT calculations and their relative stabilities were compared. Also, the effect of solvent on the isomers’ stability was predicted. The results shed the light on the importance of the intra- and inter-molecular interactions on the isomers’ stability. **4HQZN** has higher antioxidant and antibacterial activities than **4HQZ**. In contrast, **4HQZ** has higher anticancer activity against breast carcinoma MCF-7 and lung carcinoma A-549 cell lines than **4HQZN**.

## Figures and Tables

**Table 1:** Bond lengths (Å) and angles (°).

Bond Lengths			
C(1)-N(2)	1.3068(12)	C(4)-N(2)	1.3998(12)
C(1)-N(3)	1.3281(12)	C(5)-C(6)	1.3828(15)
C(2)-O(4)	1.2141(12)	C(6)-C(7)	1.3984(15)
C(2)-N(3)	1.4057(12)	C(7)-C(8)	1.3838(14)
C(2)-C(3)	1.4652(13)		
C(3)-C(8)	1.4004(13)	N(1)-O(1)	1.2470(10)
C(3)-C(4)	1.4009(13)	N(1)-O(2)	1.2530(11)
C(4)-C(5)	1.3955(13)	N(1)-O(3)	1.2622(10)
**Bond Angles**			
C(1)-N(2)-C(4)	121.44(8)	C(5)-C(4)-C(3)	121.30(9)
C(1)-N(3)-C(2)	123.88(8)	N(2)-C(4)-C(3)	118.67(8)
N(2)-C(1)-N(3)	121.89(9)	C(6)-C(5)-C(4)	118.65(9)
O(4)-C(2)-N(3)	120.17(9)	C(5)-C(6)-C(7)	120.66(9)
O(4)-C(2)-C(3)	125.69(9)	C(8)-C(7)-C(6)	120.70(9)
N(3)-C(2)-C(3)	114.14(8)	C(7)-C(8)-C(3)	119.44(9)
C(8)-C(3)-C(4)	119.24(9)		
C(8)-C(3)-C(2)	120.87(9)	O(1)-N(1)-O(2)	120.28(8)
C(4)-C(3)-C(2)	119.88(8)	O(1)-N(1)-O(3)	119.39(8)
C(5)-C(4)-N(2)	120.03(9)	O(2)-N(1)-O(3)	120.31(8)

**Table 2:** Hydrogen bonds in **4HQZN** (Å, °). ^a^

D-H…A	d(D-H)	d(H…A)	d(D…A)	<(DHA)
N(3)-H(3)…O(2)#1	0.875(15)	1.911(16)	2.7790(11)	171.2(14)
N(2)-H(2)…O(3)#2	0.914(14)	1.881(15)	2.7943(11)	176.3(13)
N(2)-H(2)…O(1)#2	0.914(14)	2.391(14)	2.9950(12)	123.6(11)
C(5)-H(5)…O(1)#3	0.95	2.36	3.1953(13)	147
C(6)-H(6)…O(2)#1	0.95	2.73	3.5690(13)	148.1
C(8)-H(8)…O(4)#1	0.95	2.5	3.4461(13)	176.6
C(1)-H(1)…O(3)#1	0.95	2.35	3.0961(12)	135.5
C(1)-H(1)…O(1)#2	0.95	2.37	2.9920(13)	123

Symmetry transformations: #1 – x + 1,y – 1/2, –z + 1/2, #2 x + 1,y,z, #3 –x + 1,y + 1/2, –z + 1/2. ^a^ DFIX command was used.

**Table 3:** The short O…H, C…O, C…N and C…C intermolecular contacts.

Contact	Distance (Å)	Contact	Distance (Å)
C2…C2	3.155	H1…O1	2.297
C4…N1	3.134	H1…O3	2.253
C1…O4	3.158	H2…O1	2.339
C2…O4	3.093	H2…O3	1.787
C3…O4	3.156	H3…O2	1.799
C1…O4	3.158	H5…O1	2.247
		H8…O4	2.364

**Table 4:** Energetics and thermodynamic parameters **^a^** of the five isomers of **4HQZN**.

Parameter	E1	E2	E3	E4	E5
**Gas**					
E	−774.161	−774.158	−774.151	−774.177	−774.15
ZPVE ^b^	0.1564	0.1566	0.1557	0.1569	0.1558
E_corr_^c^	−774.004	−774.002	−773.995	−774.02	−773.994
∆E	9.6086	11.3197	15.3745	0	16.1
H	−773.991	−773.988	−773.981	−774.006	−773.98
S	116.539	118.847	117.635	114.764	118.397
G	−774.046	−774.044	−774.037	−774.061	−774.036
**Methanol**					
E	−774.195	−774.185	−774.178	−774.189	−774.168
ZPVE ^b^	0.157	0.1548	0.1567	0.1558	0.1541
E_corr_^c^	−774.038	−774.031	−774.022	−774.033	−774.014
∆E	0	4.4565	10.1334	2.7482	14.7667
H	−774.024	−774.017	−774.008	−774.02	−774.001
S	118.292	116.599	117.834	115.358	116.384
G	−774.08	−774.073	−774.064	−774.075	−774.056
**DMSO**					
E	−774.195	−774.186	−774.179	−774.189	−774.169
ZPVE ^b^	0.157	0.1546	0.1567	0.1558	0.1539
E_corr_^c^	−774.038	−774.031	−774.022	−774.034	−774.015
∆E	0	4.4703	10.2289	3.0336	14.8717
H	−774.025	−774.018	−774.008	−774.02	−774.001
S	118.389	116.655	117.771	115.372	116.514
G	−774.081	−774.073	−774.064	−774.075	−774.056
**Cyclohexane**					
E	−774.17	−774.169	−774.158	−774.181	−774.157
ZPVE ^b^	0.1559	0.1564	0.1553	0.1565	0.1557
E_corr_^c^	−774.014	−774.012	−774.003	−774.025	−774.001
∆E	6.5339	7.8153	13.7591	0	14.7695
H	−774.001	−773.999	−773.989	−774.012	−773.988
S	116.365	117.962	116.737	114.879	117.226
G	−774.056	−774.055	−774.045	−774.066	−774.043

^a^ in a.u. except ∆E in kcal·mol^−1^ and S in cal·mol^−1^K^−1^; ^b^ zero point energy correction; ^c^ E + ZPVE.

**Table 5:** Effect of solvent on the intramolecular hydrogen bond distances in the most stable isomers **E1** and **E4**.

Medium	A ^a^	B ^a^	C ^a^	D ^a^
Gas	1.64	-	1.543	1.932
Cyclohexane	1.563	-	1.513	1.971
DMSO	-	1.664	1.462	2.023
Methanol	-	1.661	1.463	2.021

^a^ The definition of the intramolecular hydrogen bonds **A**, **B**, **C** and **D** is shown in Figure 12**.**

**Table 6:** The FMOs energies (e.v) in gas phase and different solvents.

Medium	E_HOMO_	E_LUMO_	∆E
Gas	−7.389	−2.342	5.047
Cyclohexane	−7.2673	−2.236	5.031
DMSO	−7.128	−2.499	4.630
Methanol	−7.114	−2.506	4.608

**Table 7:** Inhibition zone diameters of **4HQZ** and **4HQZN**.

Microbe	4HQZ	4HQZN	Control
*A. fumigatus*	NA	18	19 ^a^
*C. albicans*	9	10	20 ^a^
*S. aureus*	10	11	24 ^b^
*B. subtilis*	9	11	26 ^b^
*E. coli*	11	14	30 ^b^
*P. vulgaris*	11	13	25 ^b^

^a^ Ketoconazole and ^b^ gentamycin.

**Table 8:** MIC values (μg/mL) for **4HQZN** than **4HQZ**.

Microbe	4HQZ	4HQZN	Control
*A. fumigatus*	NA	312.5	156.25 ^a^
*C. albicans*	5000	2500	312.5 ^a^
*S. aureus*	2500	1250	9.7 ^b^
*B. subtilis*	2500	2500	4.9 ^b^
*E. coli*	1250	625	4.9 ^b^
*P. vulgaris*	1250	625	4.9 ^b^

^a^ Ketoconazole and ^b^ gentamycin.

## Data Availability

Not applicable.

## Supplementary Materials

Crystal structure determination; Computational DFT details; Method S1: Antimicrobial studies; Method S2: DPPH Radical Scavenging Activity; Method S3: Evaluation of Cytotoxic activity; Figure S1. FTIR spectra of 4HQZN and 4HQZ; Table S1. Crystal data for 4HQZN; Table S2. Optimized bond distances and angles of the 4HQZN (isomer E1) compared to the experimental X-ray structure results ^a^; Table S3. Natural charges at the different atomic sites of 4HQZN (isomer E1) ^a^; Table S4. Evaluation of Antioxidant Activity using DPPH scavenging assay for 4HQZ; Table S5. Evaluation of Antioxidant Activity using DPPH scavenging assay for 4HQZN; Table S6. Evaluation of cytotoxicity against Breast carcinoma MCF-7 cell line for 4HQZ; Table S7. Evaluation of cytotoxicity against Breast carcinoma MCF-7 cell line for 4HQZN; Table S9. Evaluation of cytotoxicity against lung carcinoma A549 cell line for 4HQZN.

## Sample Availability

Samples of the compounds are available from the authors.
