# Supplementary material for: Synthesis, X-ray Structure, Hirshfeld, DFT and Biological Studies on a Quinazolinone-Nitrate Complex
Source: Molecules. 2022 Feb 6;27(3):1089. doi: 10.3390/molecules27031089 (PMC8840642; doi:10.3390/molecules27031089)
Supplement: Supplementary file 1 [file molecules-27-01089-s001.zip › molecules-1571097-supplementary.pdf]

molecules

MDPI

Supplementary data

Synthesis, X-Ray Structure, Hirshfeld, DFT and Biological Studies on the Quinazolinone-Nitrate Complex

Eman M. Fathalla[1], Mezna Saleh Altowyan[2], Jörg H. Albering[3], Assem Barakat[4], Morsy A. M. Abu-Youssef[1*], Saied M. Soliman[1*] and Ahmed M. A. Badr[1]

1. Department of Chemistry, Faculty of Science, Alexandria University, P.O. Box 426, Ibrahimia, Alexandria 21321, Egypt. Emails: Eman.nomeir@alexu.edu.eg (E.M.F.), and ahmed_badr@alexu.edu.eg (AMAB).
2. [2]Department of Chemistry, College of Science, Princess Nourah bint Abdulrahman University, P.O. Box 84428, Riyadh 11671, Saudi Arabia. msaltowyan@pnu.edu.sa (M.S.A.).
3. Graz University of Technology, Mandellstr. 11/III, A-8010 Graz, Austria. Email: joerg.albering@tugraz.at (JHA).
4. Department of Chemistry, College of Science, King Saud University, P. O. Box 2455, Riyadh 11451, Saudi Arabia. ambarakat@ksu.edu.sa (A.B.)

* Correspondence: Morsy5@AlexU.edu.eg (MAMA); and saied1soliman@yahoo.com or saeed.soliman@alexu.edu.eg (SMS).

Citation: Fathalla, E.M.; Altowyan, M.S.; Albering, J.H.; Barakat, A.; Abu-Youssef, M.A.M.; Soliman, S.M.; Badr, A.M.A. Synthesis, X-ray Structure, Hirshfeld, DFT and Biological Studies on a Quinazolinone-Nitrate Complex. *Molecules* **2022**, *27*, 1089. https://doi.org/10.3390/molecules27031089

Academic Editor: Irena Kostova

Received: 10 January 2022
Accepted: 4 February 2022
Published: 6 February 2022

Publisher's Note: MDPI stays neutral with regard to jurisdictional claims in published maps and institutional affiliations.

Copyright: © 2022 by the authors. Licensee MDPI, Basel, Switzerland. This article is an open access article distributed under the terms and conditions of the Creative Commons Attribution (CC BY) license (https://creativecommons.org/licenses/by/4.0/).

Crystal structure determination

The crystal structure of **4HQZN** was determined by using a Bruker APEX II diffractometer using graphite monochromated MoKα radiation. SADABS was used for absorption corrections and all calculations were performed using SHELXTL program package. The positions of the heavy atoms were detected by the direct methods of SHELXS. The hydrogen atoms bonded to carbon atoms are refined according to the riding model implemented in SHELX. The two hydrogen atoms in the crystal structure bonded to nitrogen were refined with DFIX restraints and with fixed thermal parameter at a value of 1.5 times the displacement parameters of the respective nitrogen atoms. The CIF deposition number is CCDC - 2130306 and can be obtained from the CCDC at https://www.ccdc.cam.ac.uk/structures. All details regarding the crystallographic measurements are included in **Table S1**.

Computational DFT details

The structures of the studied isomers shown were calculated using Gaussian 09 program. The minimum energy was verified from frequency analysis. All optimized structures exhibited positive frequencies. Gauss-View4.1 has been used to draw the structures of the optimized geometries. All the calculations were first carried out in gas phase then solvent effects were studied by the Self-Consistent Reaction Field (SCRF) theory with the Polarized Continuum Model (PCM). Frequency calculations at the B3LYP/6-31+G(d,p) optimized geometries were done to compute the thermodynamic parameters such as enthalpy (H), Gibbs free energy (G), zero point vibrational energy (ZPVE) corrections. All were used to predict the relative stability of the studied isomers. Also, the natural bond orbital analyses were performed using the NBO calculations as implemented in the Gaussian 09 package at the DFT/B3LYP level.

Method S1: Antimicrobial studies [S1-S2]

a) Tested pathogenic microbes

The antimicrobial activity of **4HQZ** and **4HQZN** was evaluated against two Gram positive bacteria ((*S. aureus* (ATCC 25923) and *B. subtilis* (RCMB015(1)NRR LB-543)), two Gram negatvie bacteria ((*E. coli* (ATCC 25922) and *P. vulgaris* (RCMB 004(1)ATCC 13315)) and two fungi ((*A. fumigatus* (RCMB 002008) and *C. albicans* (RCMB 005003(1) ATCC 10231)). Gentamycin was used as standard antibacterial agent. The samples

maintained in Brain heart infusion (BHI) at 20°C; 300 mL of each stock–culture was added to 3 mL of BHI broth. Overnight cultures were kept for 24 h at 37 °C ± 1°C and the purity of cultures was checked after 24 h of incubation. After 24 h of incubation, bacterial suspension was diluted with sterile physiological solution, for the diffusion and indirect bioautographic tests, to 108 CFU/mL (turbidity = McFarland barium sulfate standard 0.5). In case of fungi *A. fumigatus* (RCMB 002008) and *C. albicans* (RCMB 005003(1) ATCC 10231), the used medium in antagonistic activity against tested fungi is Potato Dextrose Agar, where Ketoconazole was used standard antifungal agent.

b) Agar well diffusion method

Synthetic compound was prepared at concentration 10 mg/mL dissolved in DMSO as stock solutions. Preparation of sterilized Mueller Hinton agar plates seeded with tested pathogenic bacteria occurred. The wells are done by sterilized cork borer in size 6 mm and hence 100 μL of the synthetic compound was poured in each well comparably with DMSO as control. The plates were incubated at 37°C for 24-48 h (for bacteria) and at 28°C for 48 h (for fungi). After incubation period; antimicrobial activity was determined by inhibition zones.

b) Minimum Inhibitory Concentration (MIC)

Different dilutions of the compound are inoculated with tested pathogenic microbes. After incubation period of 96 well microplate, the results are measured using microplate reader. To determine at what level the MIC endpoint is established; subculture of test samples at different concentrations occurred in nutrient agar plates.

Method S2 DPPH Radical Scavenging Activity [S3]

Freshly prepared (0.004%w/v) methanol solution of 2,2-diphenyl-1-picrylhydrazyl (DPPH) radical was prepared and stored at 10 ºC in dark place. A methanolic solution of the test compound was prepared using the same procedure. A 40 uL aliquot of the methanol solution was added to 3ml of DPPH solution. Absorbance measurements were recorded immediately with a UV-visible spectrophotometer (Milton Roy, Spectronic 1201). The decrease in absorbance at 515 nm was determined continuously, with data being recorded at 1 min intervals until the absorbance stabilized (16 min). The absorbance of the DPPH radical without antioxidant (control) and the reference compound ascorbic acid were also measured. All the determinations were performed in three replicates and averaged. The percentage inhibition (PI) of the DPPH radical was calculated according to the formula:

PI = [{(*A*C- *A*T)/ *A*C} x 100] (1)

Where *A*C = Absorbance of the control at t = 0 min and *A*T = absorbance of the sample+DPPH at t = 16 min**.** The 50% inhibitory concentration (IC_{50}), the concentration required to inhibit DPPH radical by 50%, was estimated from graphic plots of the dose response curve.

Method S3 Evaluation of Cytotoxic activity [S4]

Cell line Propagation

The cells were propagated in Dulbecco's modified Eagle's medium (DMEM) supplemented with 10% heat-inactivated fetal bovine serum, 1% L-glutamine, HEPES buffer and 50μg/mL Gentamycin. Cells were maintained at 37ºC in a humidified atmosphere with 5% CO_2 and were subcultured two times a week.

Cytotoxicity evaluation using viability assay

For cytotoxicity assay, the cells were seeded in 96-well plate at a cell concentration of $1×10^4$ cells per well in 100μl of growth medium. Fresh medium containing different concentrations of the test sample was added after 24 h of seeding. Serial two-fold

dilutions of the tested chemical compound were added to confluent cell monolayers dispensed into 96-well, flat-bottomed microtiter plates (Falcon, NJ, USA) using a multichannel pipette. The microtiter plates were incubated at 37ºC in a humidified incubator with 5% CO_2 for a period of 24 h. Three wells were used for each concentration of the test sample. Control cells were incubated without test sample and with or without DMSO. The little percentage of DMSO present in the wells (maximal 0.1%) was found not to affect the experiment. After incubation of the cells for at 37°C, for 24 h, the viable cells yield was determined by the MTT test. Briefly, the media was removed from the 96 well plate and replaced with 100 μL of fresh culture DMEM medium without phenol red then 10 μL of the 12 mM MTT stock solution (5 mg of MTT in 1 mL of PBS) to each well including the untreated controls. The 96 well plates were then incubated at 37°C and 5% CO_2 for 4 hours. An 85 μL aliquot of the media was removed from the wells, and 50 μL of DMSO was added to each well and mixed thoroughly with the pipette and incubated at 37°C for 10 min. Then, the optical density was measured at 590 nm with the microplate reader (SunRise, TECAN, Inc, USA) to determine the number of viable cells and the percentage of viability was calculated as [(ODt/ODc)]x100% where ODt is the mean optical density of wells treated with the tested sample and ODc is the mean optical density of untreated cells. The relation between surviving cells and drug concentration is plotted to get the survival curve of each tumor cell line after treatment with the specified compound. The 50% inhibitory concentration (IC_{50}), the concentration required to cause toxic effects in 50% of intact cells, was estimated from graphic plots of the dose response curve for each conc. using Graphpad Prism software (San Diego, CA. USA).

References:

[S1] Pfaller, M.A.; Burmeister, L.; Bartlett; Ghorab, M.A.; Rinaldi, M.G. *J. Clin. Microbiol.* 1988, *26*, 1437-1441.

[S2]CLSI; Clinical and Laboratory Standards Institute, 2012, Twentieth informational supplement. M100-S22. Wayne: PA.

[S3] Yen, G.C. and Duh, P.D. (1994). Scavenging effect of methanolic extracts of peanut hulls on free radical and active oxygen species, *J Agric Food Chem*, 42: 629-632.

[S4] Mosmann, T. (1983): Rapid colorimetric assay for cellular growth and survival: application to proliferation and cytotoxicity assays. J. Immunol. Methods; 65: 55-63.

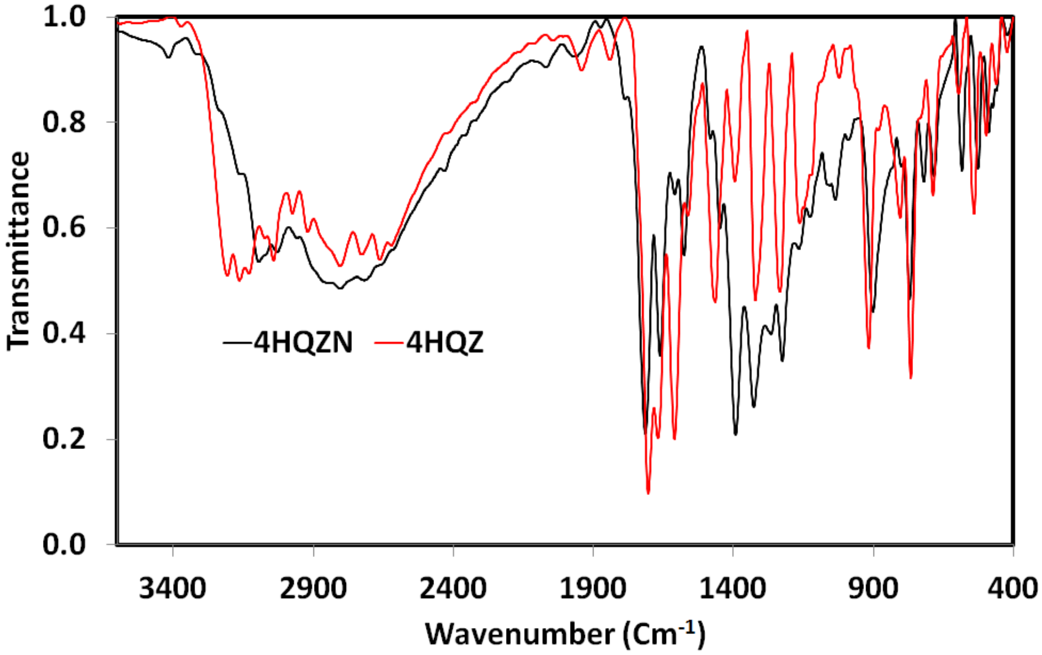

Figure S1. FTIR spectra of **4HQZN** and **4HQZ**.

Table S1. Crystal data for **4HQZN**.

CCDC	2130306	
Formula	$C_8H_7N_3O_4$	
F.Wt	209.17	
T	100(2) K	
λ	0.71073 Å	
Crystal system	Monoclinic	
Space group	$P2_1/c$	
Unit cell dimensions	a = 5.1774(3) Å	α= 90 °
	b = 10.8024(7) Å	β= 97.014(2) °
	c = 16.0958(11) Å	γ = 90 °
V	893.47(10) $Å^3$	
Z	4	
Density (calc.)	1.555 g/cm^3	
Absorption coefficient	0.128 mm^{-1}	
F(000)	432	
Crystal size	0.604 x 0.157 x 0.117 mm^3	
θ data collection	2.276 to 28.995°	
Index ranges	-7≤h≤7, -14≤k≤14, -21≤l≤21	
Reflections collected	29168	
Independent reflections	2369 [R(int) = 0.0298]	
Completeness to theta = 28.995°	100.00%	
Refinement method	Full-matrix least-squares on F^2	
Data / restraints / parameters	2369 / 2 / 143	
Goodness-of-fit on F^2	1.033	
Final R indices [I>2sigma(I)]	R1 = 0.0301, wR2 = 0.0884	
R indices (all data)	R1 = 0.0343, wR2 = 0.0946	
Extinction coefficient	0.0015(5)	
Largest diff. peak and hole	0.396 and -0.201 e.$Å^{-3}$	

Table S2. Optimized bond distances and angles of the **4HQZN** (isomer **E1**) compared to the experimental X-ray structure results[a].

Parameter	Gas	Methanol	DMSO	Chexane	X-ray
R(1-14)	1.352	1.320	1.320	1.346	1.307
R(1-16)	1.302	1.325	1.325	1.306	1.328
R(3-4)	1.485	1.469	1.469	1.481	1.465
R(3-16)	1.413	1.416	1.415	1.41	1.406
R(3-18)	1.221	1.222	1.223	1.225	1.214
R(4-5)	1.407	1.410	1.410	1.408	1.401
R(4-12)	1.403	1.404	1.404	1.404	1.401
R(5-6)	1.402	1.399	1.399	1.402	1.395
R(5-14)	1.396	1.401	1.401	1.395	1.399
R(6-8)	1.39	1.39	1.39	1.39	1.383
R(8-10)	1.405	1.406	1.406	1.406	1.399
R(10-12)	1.389	1.389	1.389	1.389	1.384
R(17-21)	1.033	**1.661**	**1.664**	1.055	**1.913**
R(16-17)	**1.640**	1.059	1.058	**1.563**	0.876
R(19-20)	1.212	1.247	1.247	1.216	1.247
R(19-21)	1.359	1.28	1.28	1.35	1.253
R(19-22)	1.234	1.26	1.259	1.234	1.262
A(14-1-16)	124.73	121.48	121.47	124.51	121.88
A(1-14-5)	121.19	122.2	122.21	121.27	121.49
A(1-16-3)	120.3	123.9	123.91	120.45	123.87
A(1-16-17)	119.21	117.46	117.47	120.17	118.09
A(4-3-16)	116.58	114.33	114.34	116.62	114.14
A(4-3-18)	123	125.9	125.91	123.24	125.69
A(3-4-5)	119.94	120.05	120.05	119.8	119.89
A(3-4-12)	121.11	120.85	120.86	121.26	120.89
A(16-3-18)	120.42	119.77	119.76	120.14	120.17
A(3-16-17)	120.49	118.64	118.62	119.38	118.03
A(5-4-12)	118.96	119.1	119.1	118.95	119.22
A(4-5-6)	121.07	121.14	121.15	121.1	121.31
A(4-5-14)	117.26	118.03	118.03	117.36	118.64
A(4-12-10)	120.33	120	120	120.29	119.45
A(6-5-14)	121.67	120.83	120.82	121.54	120.05
A(5-6-8)	118.94	118.73	118.72	118.89	118.65
A(6-8-10)	120.71	120.88	120.88	120.75	120.65
A(8-10-12)	120	120.15	120.15	120.02	120.72
A(17-21-19)	107.5	111.21	111.14	108.16	116.48
A(21-17-16)	178.5	177.49	177.67	177.07	167.86
A(20-19-21)	115.72	119.57	119.57	115.93	120.27
A(20-19-22)	126.74	121.36	121.35	126.26	119.4
A(21-19-22)	117.54	119.06	119.08	117.81	120.31

[a]Atom numbering refer to **Fig. 10**. Bold values refer to hydrogen bond interaction.

Table S3. Natural charges at the different atomic sites of **4HQZN** (isomer **E1**) [a].

Atom		Gas	Cyclohexane	DMSO	Methanol	Xray
C	1	0.3129	0.3246	0.3721	0.3717	0.4075
H	2	0.2625	0.2651	0.3005	0.3005	0.2576
C	3	0.6559	0.6618	0.6871	0.6868	0.6600
C	4	-0.1659	-0.1673	-0.1667	-0.1667	-0.1600
C	5	0.1686	0.1713	0.1762	0.1759	0.1599
C	6	-0.2666	-0.2626	-0.2429	-0.2432	-0.2193
H	7	0.2500	0.2579	0.2773	0.2769	0.2245
C	8	-0.2037	-0.2020	-0.1890	-0.1891	-0.1486
H	9	0.2572	0.2618	0.2735	0.2734	0.2245
C	10	-0.2464	-0.2449	-0.2295	-0.2295	-0.1825
H	11	0.2582	0.2620	0.2719	0.2718	0.2257
C	12	-0.1732	-0.1757	-0.1745	-0.1744	-0.1233
H	13	0.2796	0.2784	0.2795	0.2796	0.2420
N	14	-0.5787	-0.5676	-0.5213	-0.5219	-0.4811
H	15	0.4556	0.4675	0.4990	0.4985	0.4222
N	16	-0.6063	-0.6157	-0.6044	-0.6044	-0.5135
H	17	0.5220	0.5179	0.4933	0.4935	0.4603
O	18	-0.5664	-0.5939	-0.6038	-0.6023	-0.5192
N	19	0.7188	0.7211	0.7058	0.7059	0.7029
O	20	-0.3520	-0.3756	-0.5039	-0.5024	-0.4722
O	21	-0.5400	-0.5422	-0.5605	-0.5604	-0.5753
O	22	-0.4422	-0.4421	-0.5397	-0.5404	-0.5923

[a]Atom numbering refer to **Fig. 10.**

Table S4. Evaluation of Antioxidant Activity using DPPH scavenging assay for **4HQZ.**

Sample conc. (μg/mL)	DPPH scavenging %	(±)SD
1280	23.76	1.29
640	11.94	0.88
320	5.32	0.76
160	1.87	0.49
80	0.29	0.17
40	0.08	0.05
20	0.02	0.01
10	0	
5	0	
2.5	0	
0	0	

Table S5. Evaluation of Antioxidant Activity using DPPH scavenging assay for **4HQZN.**

Sample conc. (μg/mL)	DPPH scavenging %	(±)SD
1280	91.89	0.75
640	88.71	1.03
320	83.26	0.92
160	78.43	1.21
80	60.46	1.78
40	53.80	2.04
20	31.47	2.39
10	16.03	1.41
5	9.48	0.64
2.5	4.92	0.36
0	0	

Table S6. Evaluation of cytotoxicity against Breast carcinoma MCF-7 cell line for **4HQZ**.

Sample conc. (μg/mL)	Viability %	Inhibitory %	S.D. (±)
500	19.28	80.72	2.04
250	37.06	62.94	2.98
125	59.63	40.37	1.75
62.5	80.41	19.59	1.37
31.25	97.65	2.35	0.79
15.6	100	0	
7.8	100	0	
3.9	100	0	
2	100	0	
1	100	0	
0	100	0	

Table S7. Evaluation of cytotoxicity against Breast carcinoma MCF-7 cell line for **4HQZN**.

Sample conc. (μg/mL)	Viability %	Inhibitory %	S.D. (±)
500	28.06	71.94	1.72
250	49.32	50.68	2.68
125	86.53	13.47	1.89
62.5	98.12	1.88	0.64
31.25	100	0	
15.6	100	0	
7.8	100	0	
3.9	100	0	
2	100	0	
1	100	0	
0	100	0	

Table S8. Evaluation of cytotoxicity against lung carcinoma A549 cell line for **4HQZ**.

Sample conc. (μg/mL)	Viability %	Inhibitory %	S.D. (±)
500	13.95	86.05	0.67
250	29.43	70.57	1.29
125	48.21	51.79	2.45
62.5	72.97	27.03	3.11
31.25	88.06	11.94	0.82
15.6	98.12	1.88	0.64
7.8	100	0	
3.9	100	0	
2	100	0	
1	100	0	
0	100	0	

Table S9. Evaluation of cytotoxicity against lung carcinoma A549 cell line for **4HQZN**.

Sample conc. (μg/ml)	Viability %	Inhibitory %	S.D. (±)
500	24.69	75.31	1.73
250	46.34	53.66	2.06
125	81.96	18.04	1.88
62.5	97.89	2.11	0.97
31.25	100	0	
15.6	100	0	
7.8	100	0	
3.9	100	0	
2	100	0	
1	100	0	
0	100	0	
